# *Drosophila melanogaster* larvae control amylase secretion according to the hardness of food

**DOI:** 10.3389/fphys.2013.00200

**Published:** 2013-08-13

**Authors:** Honami Sakaguchi, Masataka G. Suzuki

**Affiliations:** Department of Integrated Biosciences, Graduate School of Frontier Sciences, The University of TokyoKashiwa, Japan

**Keywords:** external digestion, *Drosophila melanogaster*, larvae, amylase, secretion

## Abstract

*Drosophila melanogaster* larvae excrete amylase and perform external digestion of their food. In this study, to investigate whether their external digestion ability varies in response to changes in the external environment, we measured the relative amount of amylase excreted by larvae using a new method: the iodine starch agar method (ISAM). Analysis using this method revealed that the amount of amylase excreted by larvae increased in accordance with the increase in the agar concentration. In addition, we investigated the effect on the larval growth rate of adding amylase to the diet. Pupation occurred 24 h later in food containing 1% amylase than in food containing no amylase. These results suggest that the larvae adjust their amylase excretion in response to changes in the external environment, and that its level has a marked influence on the larval growth rate.

## Introduction

Humans digest food after putting it into their mouths. Surprisingly, bacteria and some animals such as *Octopus vulgaris* paralarvae, Pogonophora, and *Drosophila melanogaster* larvae perform digestion by excreting enzymes into the external environment (Ivanov, 1955; Gregg et al., 1990; Vicente et al., 2000; Bonner, 2001). Notably, *D*. *melanogaster* larvae facilitate external digestion by associating in clusters and producing more digestive enzymes. This phenomenon is called “social digestion” (Gregg et al., 1990). However, the purpose, regulation, and functional significance of external digestion have yet to be determined. Many “Drosophilists” have noticed that the surface of an artificial diet gradually becomes wet and softened as the larvae grow. Accordingly, we predicted that larvae excrete digestive enzymes such as amylase, which physically soften the food and improve the efficiency of food ingestion. Moreover, food softening seems to help larvae to move around in the food.

To test this hypothesis, we developed the iodine starch agar method (ISAM), which enabled us to estimate the amount of amylase secreted into agar. Moreover, we investigated how addition of amylase to the diet affects the larval growth rate. Here, we report that the larvae adjust their amylase excretion in response to changes in the external environment, and that the amount of amylase added to the food medium has a marked influence on the larval growth rate.

## Materials and methods

### Drosophila strains

In all experiments, we used Canton-S *Drosophila melanogaster* larvae, maintained at 25°C on Carolina Biological Instant Drosophila Medium (Formula 4–24) with 0.04 g of live yeast, according to a protocol described previously (Kawasaki et al., 2007).

### Detection of amylase

**Figure 1:**
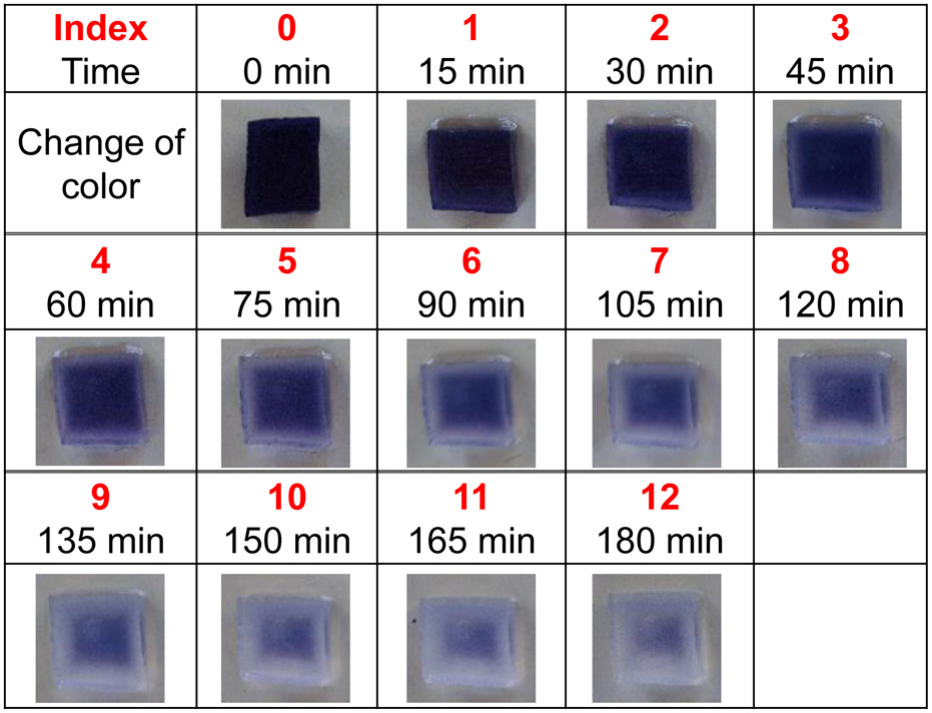
**Gradual digestion of starch after application of amylase**. The change in color was recorded every 15 min (for a total of 180 min) and color intensity was indexed into 12 levels.

To estimate the relative amount of secreted amylase, a new method (ISAM) was developed. This method can be used to establish the color intensity associated with various levels of starch after adding a set amount of amylase. Amylase digests starch, and starch can be quantified based on the amount of color produced by the iodo-starch reaction (Xiao et al., 2006). In ISAM, an agar cube (1.5 × 1.5 × 1.5 cm) containing 5% starch, 10% sucrose, and 0.5% iodine was prepared. The concentration of agar in each cube ranged from 0.05 to 1.5%, yielding cubes with different hardness levels. Twenty second-to-third instar larvae were placed on each cube at room temperature. Because there is a negative correlation between the amount of amylase and the intensity of agar color at a given time, the relative amount of amylase was estimated roughly by the color grade of the agar cube. The fading of agar color was examined and recorded by photography. To evaluate the level of digestion, 15 mg of amylase was applied to the agar cube, and then the color intensity was indexed into 12 levels by photographing the agar cube every 15 min after the amylase application, as shown in Figure 1. An index value of 0 represents no digestion and no amylase. Using this index, the relative amount of amylase excreted from larvae can be described. Under our experimental conditions, the substrate concentration was sufficiently high. This means that the rate of digestion of starch by amylase was constant. Under these conditions, the relative amount of amylase excreted from larvae was estimated using the following equation: [reaction time] × [amount of amylase] = [amount of digested starch]. If a *T* (min) reaction of *X* mg amylase with the agar cube results in the color index *Y*, then *X* can be calculated from the following equation: *X* = [Y × 15 min × 15 mg]/*T* (min). In this equation, 15 min represents the interval time between the color indices shown in Figure 1, and 15 mg represents the amount of amylase used to establish the color index.

### Measurements of pupation rate in amylase-supplemented groups

Adult flies aged 4–5 days were used. Five pairs of flies were transferred into a vial containing a fresh artificial diet and were allowed to lay eggs for 1 day. Adult flies were then removed, and 500 μl of distilled water containing amylase (1, 10^−2^, 10^−3^, or 10^−4^%, v/w) was added to each vial. A volume of 500 μl of MQ water without amylase was added to vials as a negative control. The number of pupae was counted once per day for 14 days after egg laying. The pupation rate was calculated from the total number of pupae and the larval growth rates were compared.

### Statistical analysis

Significant differences between the groups receiving different treatments were identified with an unpaired Student's *t*-test. The experiments in Figures 6–8 were repeated five times. The error bars in the graphs indicate the standard error of the mean. Significance was accepted at *p* < 0.05.

## Results

### Larvae regulate amylase secretion in response to the hardness of food

**Figure 2:**
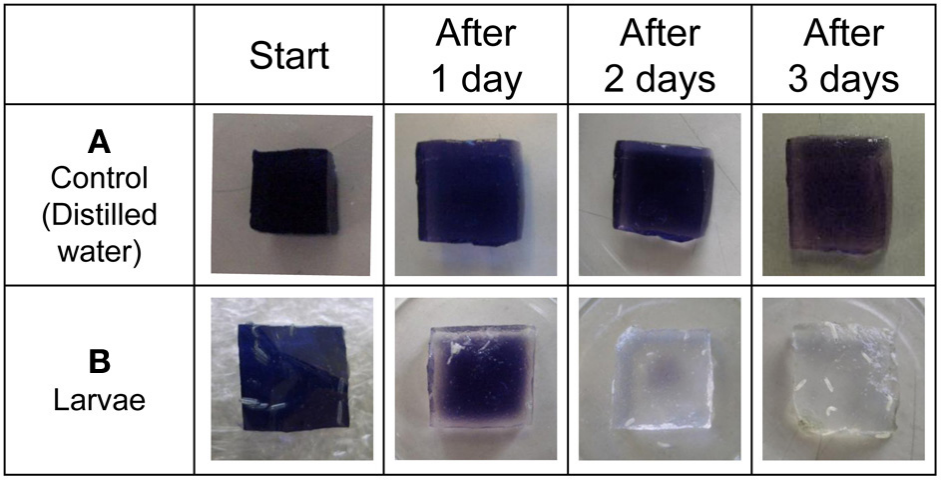
**Detection of amylase excreted from larvae by ISAM**. Distilled water **(A)**, and fruit fly larvae **(B)** were applied to each gel cube (1% agar, 5% starch, 0.5% iodine, 10% sucrose), and the cubes were incubated for 3 days at room temperature. The color fading of each gel was examined daily and photographed. The control gel did not change color, while larvae caused gradual color fading of gels. The color fading clearly showed that fruit fly larvae secreted saliva from their mouths.

As a first step, we confirmed whether fruit fly larvae secrete amylase on food medium. The blue-colored iodinated agar medium was exposed to either distilled water or fruit fly larvae. The blue color faded gradually 2 or 3 days after exposure to fruit fly larvae (Figure 2). Consistent with previous reports, these results demonstrate that fruit fly larvae secrete amylase onto the food medium under our experimental conditions.

**Figure 3:**
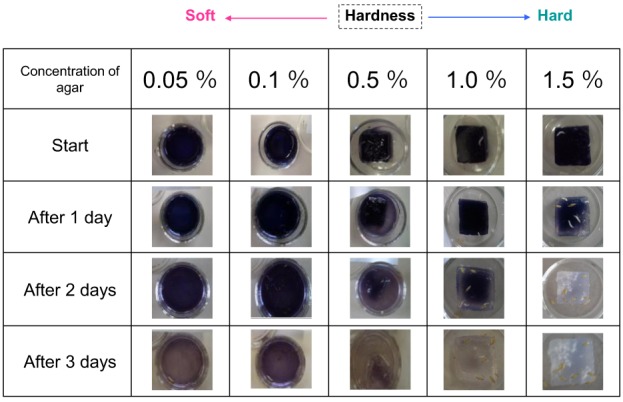
**Relationship between agar cube hardness and the amount of amylase excreted**. Gels containing different amounts of agar (0.05–1.5%) were used in ISAM. The color fading of each gel was examined for 3 days and photographed. The color of harder gels faded more rapidly, indicating that larvae excreted more saliva on harder gels.

Next, to investigate whether larvae increase amylase secretion in response to an increase in food hardness, larvae were placed on blue-colored iodinated agar medium of different degrees of hardness. The color faded more rapidly as the agar concentration in the medium increased (Figure 3). A similar tendency was observed when the experiments were replicated. These results indicate that the larvae increased amylase secretion when the medium became harder.

**Figure 4:**
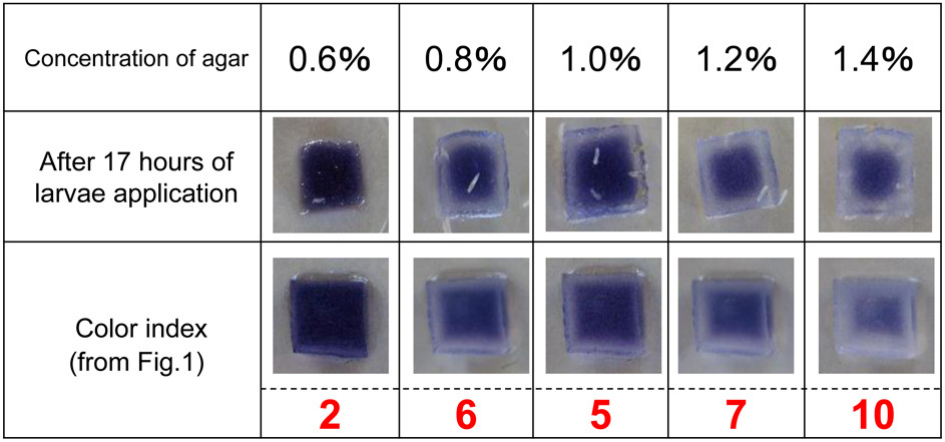
**Level of starch digestion 17 h after larval application**. The rate of starch digestion was examined by changing the concentration of agar used in the ISAM from 0.6 to 1.4% at 0.2% intervals. Twenty larvae were applied simultaneously to each gel. The color of gel pieces faded more rapidly with harder gels. The change in gel color at 17 h after larvae application was photographed and the color fading was compared with that in Figure 1. The value of the color index corresponding to the same gel color (the same level of color fading) was chosen to evaluate the relative amount of amylase in each gel.

**Figure 5:**
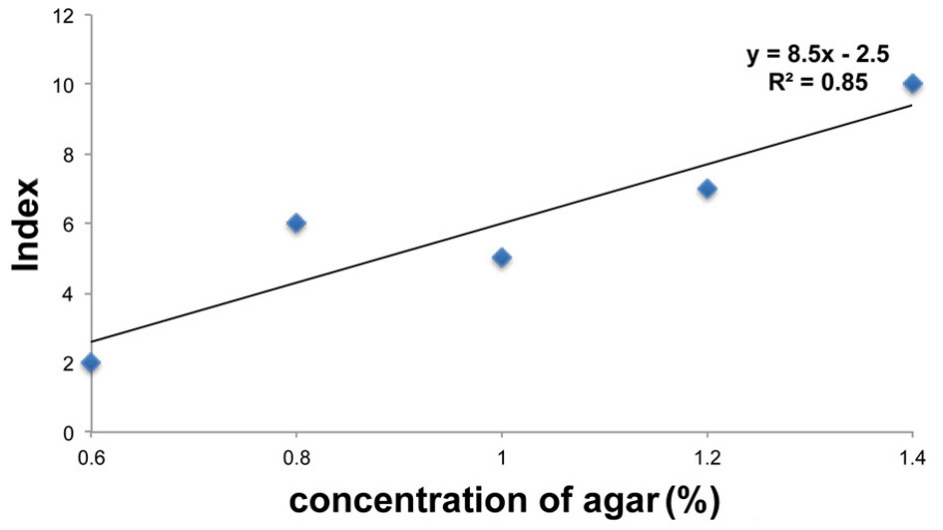
**Relation between food hardness and the amount of amylase**. The values of the color index in each agar concentration are plotted on the graph. The vertical axis represents the color index and the horizontal axis represents the concentration of agar. The value of the color index increased with a concomitant increase in the agar concentration.

Color fading of agar cubes was indexed and correlated with the grades of hardness of the medium. Using this color-fading index, we determined the color fading levels of each medium with different agar concentrations at 17 h after larva application (Figure 4). As shown in Figure 5, there was a linear relationship between the hardness of the medium and the index of color fading. These results indicated that the amount of amylase excreted by larvae was increased in accordance with the increase in the agar concentration. On the basis of the equation described in the Materials and Methods, we expected that a single drosophila larva would excrete saliva with an amylase activity comparable to ~1 μg of amylase as the agar concentration increased by 0.1%.

### Increasing the amylase content of the food media delayed the pupation peak

**Figure 6:**
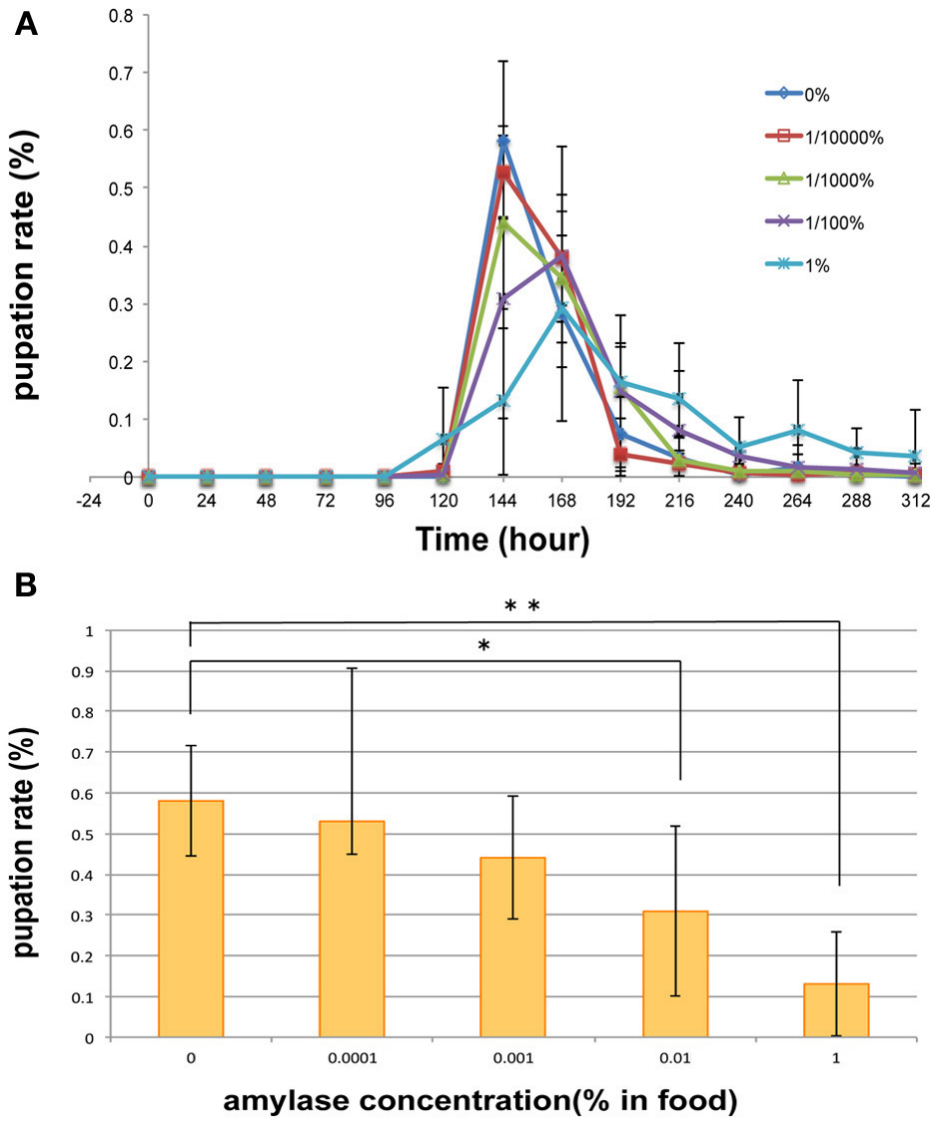
**Effect on pupation rate of addition of amylase to the diet**. **(A)** For diets supplemented with 0, 10^−4^, and 10^−3^% amylase, pupation peaked at 144 h after egg laying. Supplementation of the diet with 10^−2^ or 1% amylase delayed the pupation peak for 24 h. The pupation peak occurred later as more amylase was added to the food. **(B)** At 144 h after egg laying, a significant difference was detected between the 0 and 1% amylase-supplemented groups (*p* < 0.01), and between the 0 and 10^−2^% amylase-supplemented groups (*p* < 0.05). ^*^ indicates *p* < 0.05. ^**^indicates *p* < 0.01.

**Figure 7:**
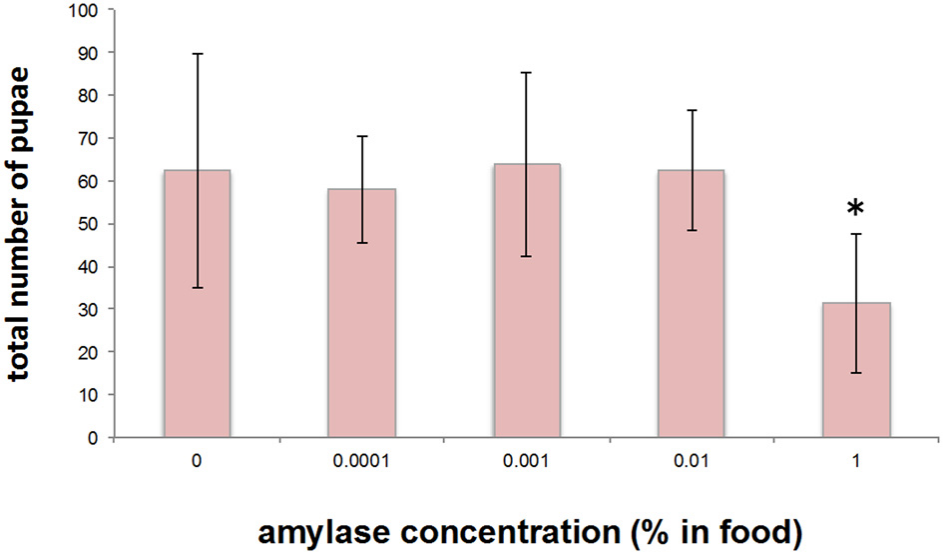
**Effect on the total number of pupae of addition of amylase to the diet**. The total number of pupae did not differ significantly between the groups, except for the 1% amylase-treated group. ^*^indicates *p* < 0.05.

In an attempt to gain an insight into the biological importance of the secretion of amylase into the food medium, we investigated the effect of adding amylase to diet on the larval growth rate. For this purpose, various concentrations of amylase (10^−2^, 10^−3^, and 10^−4^%, v/w) were added to the fresh food medium of each vial in which adults laid eggs for 24 h (see Materials and Methods). As more amylase was added to the food, pupation peaked later (Figure 6). We suppose that the larval period was prolonged to actively ingest nutrients from the softened food because addition of amylase enhanced external digestion by larvae. There was no significant difference in the total number of pupae between the control and amylase groups (Figure 7). These results indicate that addition of amylase did not affect the larval survival rate. However, the total number of pupae was significantly lower in the 1% amylase-treated group. This might be because many larvae could not pupate during the test period (2 weeks).

**Figure 8:**
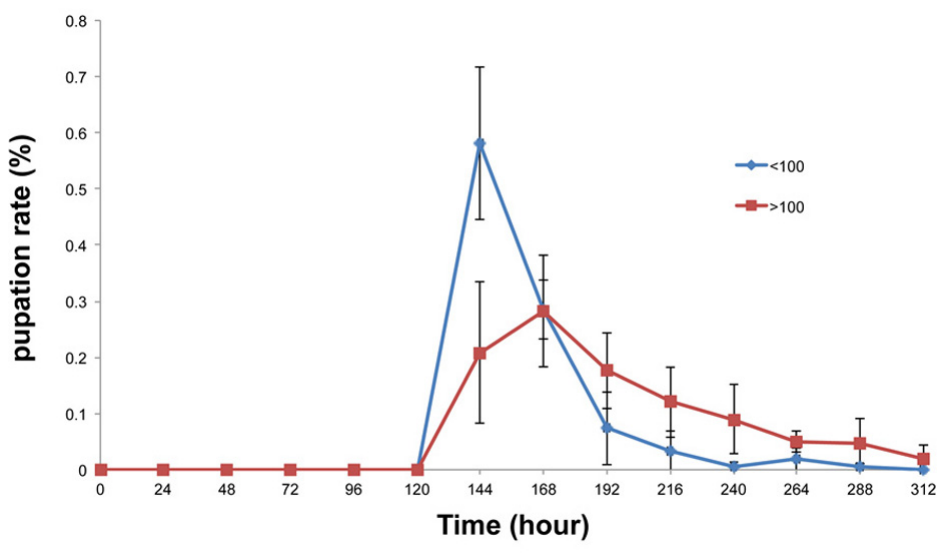
**Comparison of the pupation rates of the large and small population groups**. Pupation peaked 24 h later in the large population group (more than 100 larvae) than in the small population group (fewer than 100 larvae). At 144 h after egg laying, a significant difference was detected between the groups with more than 100 pupae and less than 100 pupae (*p* < 0.01).

It might be expected that the total amount of amylase secreted into the food medium would be increased in a large population. If this were so, then the condition of the food medium would become similar to that of the amylase-supplemented medium. To test this hypothesis, the pupation peak was compared between large and small populations. Consistent with our expectations, pupation was delayed when the total number of pupae per vial was more than 100, as was the case in the amylase-treated group (Figure 8).

## Discussion

Amylase gene expression is known to be repressed and amylase activity reduced by glucose (Hickey and Bernhard, 1982; Benkel and Donal, 1986). Hickey and Bernhard reported that amylase activity was reduced by dietary glucose in *Drosophila* adults and larvae. Moreover, it was suggested that the level of amylase mRNA was decreased in the presence of glucose.

In this study, we revealed that the amount of amylase excreted from *Drosophila* larvae was affected by another factor: the hardness of the food (the concentration of agar). As the hardness of the food was progressively increased, more amylase was excreted (Figure 3), suggesting that larvae increase their amylase excretion in response to food hardening. This strongly suggests that *Drosophila* larvae have the ability to control the excretion of amylase, rather than simply egesting it, and thus actively regulate external digestion.

Our results suggest that increasing the amylase content of the food medium had the same effect on the larval growth rate as that observed in the large population group. We found that pupation was delayed by the addition of amylase to the food (Figure 6). It is conceivable that the addition of amylase to the food increases its glucose content, which in turn may have caused the pupation delay. Consistent with this hypothesis, glucose-enriched food delays pupation in *Plodia interpunctella* (Bouayad et al., 2008). Bouayad reported that amylase activity was reduced when the larvae were grown in glucose-enriched food. Moreover, their results showed that the glucose-enriched food delayed pupation, increased body weights, and decreased mortality. We speculate that larvae actively ingest nutrients from the softened food to increase their body weights because the added amylase enhanced external digestion. Delayed pupation was also observed in the presence of large numbers of pupae (Figure 8). It is expected that the amount of amylase secreted into the food medium would be increased in more or less direct proportion to the number of larvae. Therefore, the condition of the food could be similar to that of the amylase-supplemented media. This may have caused the pupation delay observed in the large population group.

In conclusion, based on the experiments described above, we believe that *Drosophila* larvae adjust their amylase excretion in response to changes in the external environment to make it more suitable for their growth. To fully understand the biological importance of amylase excretion, further research is needed to clarify the relationship between amylase secretion and larval weight, larval mortality, and adult fertility.

### Conflict of interest statement

The authors declare that the research was conducted in the absence of any commercial or financial relationships that could be construed as a potential conflict of interest.
